# Supplementary material for: Assessment of Physicians’ Willingness to Work with Patients Not Yet Diagnosed with COVID-19 in a Romanian Sample
Source: Healthcare (Basel). 2024 Jan 10;12(2):161. doi: 10.3390/healthcare12020161 (PMC10815129; doi:10.3390/healthcare12020161)
Supplement: Supplementary file 1 [file healthcare-12-00161-s001.zip › healthcare-2710666-supplementary.pdf]

Supplementary S1 List of All Study Variables and Questions

Unique subject number for database control

01. Gender of participating healthcare professional

02. Age of healthcare professional in years

03. What region do you work in?

04. Area where your practice

05. What is your marital status?

06. How many people do you currently live with?

07. Do you currently live with a family member?

08. How many children/youth between the ages of 6 and 18 live with you?

09. Number of children under the age of 5 living with you?

10. How many people over the age of 65 currently live with you?

11. How many rooms do you have in your home?

12. Is there any other source of income in your household besides your health care professional's salary?

13. On a scale of 1 to 10, how would you rate your economic status?

14. Are you currently employed?

15. Did you lose your job as a result of the COVID-19 pandemic?

16. Do you have a source of income?

17. How much have you suffered economically since the pandemic?

18. What type of work do you do?

19a Healthcare Worker Self-reported Ischemic/Hemorrhagic/Transient Stroke

19b Healthcare Worker Self-reported Retinopathy

19c Health professional self-reported Myocardial infarction/angina pectoris/coronary heart disease

19d Healthcare Professional Self-reported Emphysema or Chronic Obstructive Pulmonary Disease (COPD)

19e Healthcare Professional self-reported type 1 diabetes (one)

19f Healthcare Professional self-reported chronic diarrheal disease

19g Healthcare Professional self-reported neoplasia

19h Healthcare Professional self-reported chronic constipation

19i Healthcare Professional self-reported osteoarthritis

19j Healthcare Professional self-reported cataract

19k Healthcare Professional self-reported hypertension

19l Healthcare Professional self-reported trauma

19m Healthcare Professional Self-reported Chronic Low Back Pain

19n Healthcare Professional Self-reported Chronic Neck Pain

19o Healthcare Professional Self-reported Urinary Incontinence or Incontinence Control Disorder

19p Healthcare Professional Self-reported Inflammatory Bowel Disease

19r Healthcare Professional self-reported migraine/chronic headache

19s Healthcare Professional self-reported chronic abdominal pain

19t Healthcare Professional self-reported bronchial asthma (including allergic asthma)

19u Healthcare Professional self-reported chronic allergies (excluding allergic asthma)

19v Healthcare Professional self-reported kidney disease

19x Healthcare Professional Self-reported Peptic Ulcer

19z Healthcare Professional Self-reported Obesity

19aa Healthcare Professional Self-reported Thyroid Disease

19ab Healthcare Professional Self-reported Varicose Veins of Lower Limbs

19ac Healthcare Professional Self-reported Dyslipidemia

19ad Healthcare Professional Self-reported Chronic Dermatologic Conditions

19ae Healthcare Professional Self-reported Type 2 Diabetes (Two)

19af Healthcare Professional self-reported hemorrhoids

19ag Healthcare Professional Self-reported Osteoporosis

19ah Healthcare Professional Self-reported Liver Disease

19ai Healthcare Professional self-reported at least one chronic disease

20. Over the past two weeks, how would you rate your physical health?

21. But in the last two weeks BEFORE the COVID-19 pandemic?

22. Your role as a healthcare professional

23. Level of education (choose the highest level of education you have completed)

24. Specialty of health professional

25. Type of practice

26a. Health professional self-identifying as working in MATERNITY

26b. Health professional self-identifying as working in PALLIATIVE SERVICES

26c. Health professional self-identifying as working in SURGICAL SERVICES

26d. Health professional self-identifies as working in NURSING HOME / TERMINAL

26e. Health professional self-identifies as working in MEDICAL SECTION

26f. Health professional self-identifies as working in INTENSIVE THERAPY

26g. Health professional self-identifies as working in EMERGENCY/AMBULANCY DEPARTMENT

26h. Health professional self-identifies as working in DAILY SPITALIZATION

26i. Health professional self-identifies as working in FAMILY MEDICINE

26j. Health professional self-identifies as working in CLINICAL OFFICE

27. Years in practice

28. On average, how many hours per week did you work during the pandemic period?

29. Do you think it is possible to have been in the same room as a COVID-19 positive person?

30. Please indicate the exact number of confirmed cases you have been in contact with.

31. How many of the confirmed COVID-19 patients you have worked with have died?

32. How many times have you been tested for COVID-19 infection?

33. Have you been hospitalized because of COVID-19 infection?

34. Has anyone close to you been hospitalized because of COVID-19 infection?

35. Has anyone close to you died because of COVID-19 infection?

36. Have you worked in a COVID-19 quarantined area?

37a. Healthcare worker reports that he/she has not been involved in any of the activities listed.

37b. Healthcare worker reports collecting biological samples from COVID-19 suspects.

37c. Healthcare worker reports handling respiratory samples

37d. Healthcare worker reports screening without direct contact with patients

37e. Healthcare worker reports having performed aerosol-generating procedures

37f. Healthcare worker reports direct contact with COVID-19 patients

37g. Healthcare worker reports examining patient WITH respiratory symptoms

37h. Healthcare worker reports seeing patient WITHOUT respiratory symptoms

38. It would have been acceptable for health care workers to leave their jobs during the pandemic to protect themselves and their families. DUTY [Reversed]

39. What do you think of the idea of allowing a health professional to decide whether to come to work during a pandemic? DUTY

40. How do you feel about dismissing those who did not work during the pandemic when they could have? DUTY

41. What do you think about the criminalization of those who did not go to work during the pandemic when they could have? DUTY

42. What do you think about the extent of the loss of the right to practice for those who did not practice during the pandemic, although they could have? DUTY

43. Should a health professional who does not have children work on the front line during a pandemic? DUTY

44. A health professional who does not have a chronic disease should work on the front line during a pandemic. DUTY [Reversed]

45. A young healthcare professional should work on the front line during a pandemic. DUTY [Reversed]

46. A healthcare worker with risk factors for severe progression of COVID-19 should work on the front line. [DUTY]

47. It seemed very natural to work during the pandemic. [DUTY]

48. When I was asked to work with patients with COVID-19, I was ready to respond.

49.I was aware of my role during the pandemic. [AGENCY] [SELF-EFFICACY].

50.I had the skills for my role during the pandemic. [AGENCY] [SELF-EFFICACY]

51.I was willing to provide direct patient care even if I did not have access to overalls, although I should have used them. [AGENCY] [THREAT].

52. I was willing to provide direct patient care when I did not have access to gloves when I should have used them [AGENCY] [THREAT].

53.I was willing to provide direct patient care when I did not have access to the K95 mask when I should have used it [AGENCY] [THREAT].

54.I was willing to provide direct patient care despite not having access to protective equipment when I should have used protective equipment [AGENCY] [THREAT].

55.I felt psychologically prepared to work during the pandemic [AGENCY] [SELF-EFFICIENCY].

56. It was easy for me to safely use transportation to work during the pandemic. [AGENCY] [THREAT]

57. I was confident that I would be safe at work during the pandemic. [AGENCY] [THREAT]

58.My family was prepared to manage without me while I worked during the pandemic. [AGENCY] [SELF-EFFICACY]

59. My role in the overall response to the pandemic was important. [AGENCY] [SELF-EFFICACY]

60. It would have been more frustrating for me to stay home and not be able to work, knowing that I could contribute.

61. In my job, you don't just work when things are rosy. [AGENCY] [AVAILABILITY]

62. Because of peer pressure, I took the same risks as my colleagues.

63. I worked because I could leave work when I needed to. [ASSUMING THE CONSEQUENCES].

64. I worked because my colleagues also reported for duty.

65. I worked even though no one would report me if I left the program [AGENCY].

66. I took the same risks as my colleagues for the good of the community. [ASSUMING CONSEQUENCES].

67. I took the same risks as my colleagues because people expected us to. [ASSUMING CONSEQUENCES].

68.I took the same risks as my colleagues because people trusted us [ASSUMING CONSEQUENCES].

69.I took the same risks as my colleagues because I am committed to helping those in need through my profession. [ASSUMING CONSEQUENCES AS A RESULT OF PROFESSIONAL VALUES].

70. Not coming to work during a pandemic is letting your team down. [ASSUMING CONSEQUENCES].

71.Not coming to work during a pandemic means abandoning your patients. [ASSUMING CONSEQUENCES].

72.I worked during the COVID-19 pandemic even though it meant a higher risk of infecting my family than the usual risk [AVAILABILITY].

73. I was willing to work during the pandemic even though I was asked to take on responsibilities for which I was not prepared [AVAILABILITY].

74. I was willing to work during the pandemic even if I was asked to work overtime [AVAILABILITY].

75. I was willing to work during the pandemic even if schools, kindergartens, or nurseries were closed [WILLINGNESS TO WORK].

76.I was willing to work during the pandemic even if my partner was infected with COVID-19 [WILLINGNESS TO WORK].

77. I was willing to work during the pandemic even if my child/children got COVID-19 [WILLINGNESS TO WORK].

78. I was willing to work during the pandemic even if my parents had COVID-19 [WILLINGNESS TO WORK].

79. I was willing to work during the pandemic even if I was asked to work in a different hospital or place than usual [WILLINGNESS TO WORK].

80. I was willing to work during the pandemic even if I was asked to discharge patients from the hospital early [WILLINGNESS TO WORK].

81. I was willing to work during the pandemic even if my COVID-19 colleagues died.

82.I was willing to work during the pandemic even if my relatives died. [WILLINGNESS TO WORK].

83.I was willing to work during the pandemic even if the people I live with were infected by me. [WILLINGNESS TO WORK].

84. Healthcare professionals have a duty to patients, despite the risks.

85. All health professionals have a duty to work, even if the risk is very high.

86. Everyone must help during the pandemic. [SOLIDARITY]

87.Healthcare workers should have the right to refuse to work with infected patients, even if they have all the necessary protective equipment. DUTY [Reversed]

~~88.Healthcare workers should have the right to refuse to work with infected patients, even if they do NOT have all the necessary protective equipment. [excluded].~~

89.Healthcare workers should lose their pay if they don't report to work during the pandemic. [DUTY]

90. Health professionals who work during the pandemic should be paid extra. DUTY [Reversed]

91.The financial bonus we were promised justifies the risk I took. DUTY [Reversed]

92.My main responsibility is to myself and my family. [Responsibility to family and self, single item].

93.I had to work because I was dependent on my salary. [CONSEQUENCES OF PRESSURE].

94.We had access to the necessary protective equipment.

95.I worked during the pandemic because I was afraid of losing my job. [ASSUMING CONSEQUENCES THROUGH PRESSURE].

96.I worked during the pandemic because I was afraid of legal repercussions against me [ASSUMING CONSEQUENCES THROUGH PRESSURE].

97. I worked out of loyalty to my patients. [ASSUMING CONSEQUENCES FROM PROFESSIONAL VALUES].

98.My work during the pandemic put me at risk of malpractice.

99a Infection Control Procedures Negatively Impacted Medical Practice (Hierarchy)

99b Canceling non-emergency appointments negatively influenced medical practice (hierarchy)

99c Fear of keeping patients out of hospital negatively influenced practice (hierarchy)

99d Restrictions on hospital visitors negatively influenced practice (hierarchy)

99e Additional duties not mentioned in job description negatively influenced medical practice (hierarchy)

99f Poor communication of COVID-19-related guidelines negatively influenced practice (hierarchy)

99g Clarification of patients' ideas about preventive measures negatively influenced medical practice (hierarchy)

99h Problems concentrating at work negatively influenced medical practice (hierarchy)

99i Discharge of non-urgent patients from hospital negatively influenced medical practice (hierarchy)

99j Staff burnout has negatively influenced medical practice (hierarchy)

100. I was trained to use the protective equipment used during the pandemic.

101. The management of the organization where I work has made every effort to provide sufficient personal protective equipment and necessary disinfectants. [INSTITUTIONAL RECIPROCITY].

102. There was clear, continuous, transparent and honest communication with all employees about the risks of the pandemic [INSTITUTIONAL RESPONSIBILITY].

103. The institution where I work had a pandemic plan that was continuously updated [INSTITUTIONAL RESPONSIBILITY].

104. In the institution where I work, there were people with the necessary competence to answer any questions I had about my personal safety or the safety of my family. [INSTITUTIONAL RECIPROCITY].

105. How much trust do you have in the management of the institution where you work? [TRUST IN THE INSTITUTION]

106. The management of my institution has communicated a SARS-COV-2 infection control plan to all employees...[INSTITUTIONAL RECIPROCITY].

107. The institution's management has openly and continuously demonstrated that it has taken responsibility for protecting and supporting staff during the pandemic. [INSTITUTIONAL RECIPROCITY].

108. The institution's management ensured that I was adequately compensated during the crisis. [INSTITUTIONAL RECIPROCITY]

109. Those who run the institution where I work have made every effort to make employees feel valued. [INSTITUTIONAL RECIPROCITY]

110. Any changes in job responsibilities or expectations have been clearly described. [INSTITUTIONAL RECIPROCITY]

111. The management of the institution where I work has shown understanding for the problems I have raised and has supported me [INSTITUTIONAL RECIPROCITY].

112. The management of the institution has shown respect in communicating with me. [INSTITUTIONAL RECIPROCITY]

113.The management of the institution has shown respect in communicating with all employees. [INSTITUTIONAL RECIPROCITY]

114. Complying with the rules imposed by the pandemic in my workplace was too disruptive. [INSTITUTIONAL RECIPROCITY] [R]

115. The rules at my workplace were not strict enough [INSTITUTIONAL RECIPROCITY] [R].

116. Our SARS-COV-2 infection control rules were more reasonable than those of other similar institutions. [INSTITUTIONAL RECIPROCITY].

117. My employer and I share the same values. [values shared with institution - single variable].

118. During the pandemic, my employer was concerned about the safety of my family. [INSTITUTIONAL RECIPROCITY].

119. During the pandemic, my employer was concerned about my ability to get to work. [INSTITUTIONAL RECIPROCITY]

120. I felt I could raise public health issues with the relevant institutions. [TRUST IN GOVERNMENT].

121.I was aware of the public health implications of the pandemic. [AGENCY] [SELF-EFFICACY]

122. The information we received from the Directorate of Public Health about the risks of SARS-COV-2 infection was timely. [GOVERNMENT RECIPROCITY].

123. The information I received from the Directorate of Public Health about my professional role was complete and accurate. [GOVERNMENT RECIPROCITY].

124. The information I received from the Directorate of Public Health was based on scientific evidence and guided my medical work. [STATE RECIPROCITY].

125. Any changes in job responsibilities or expectations were clearly described. [GOVERNMENT RECIPROCITY]

126. The DSP showed respect when communicating with me. [STATE RECIPROCITY] 127.

127.The DSP showed respect in communicating with my colleagues. [GOVERNMENT RECIPROCITY]

129a. Sources of information during the pandemic: MY DIRECTOR (Hierarchy)

129b. Sources of Information During the Pandemic: HOSPITAL ADMINISTRATION (Hierarchy)

129c. Information sources during the pandemic: COLLEAGUES IN OTHER WARDS OF THE HOSPITAL (hierarchy)

129d. Information sources during the pandemic: OTHER COMMUNITY COLLEAGUES (hierarchy)

129e. Information sources during the pandemic: COLLEAGUES AT WORK (hierarchy)

129f. Information sources during the pandemic: PROFESSIONAL SOCIETIES OR ORGANIZATIONS IN ROMANIA (hierarchy)

129g. Information sources during the pandemic: PROFESSIONAL ORGANIZATIONS ABROAD (hierarchy)

129h. Sources of information during the pandemic: HEALTH MINISTRY SPECIALISTS (hierarchy)

129i. Information sources during the pandemic: ONLINE MEDIA INFORMATION GROUPS (hierarchy)

129j. Information sources during the pandemic: OTHER SOURCES (hierarchy)

130a. Moral or personal support when I had a professional problem: MY DIRECT SUPERVISORS (hierarchy)

130b. Moral or personal support when I had a professional problem: HOSPITAL ADMINISTRATION (hierarchy)

130c Moral or personal support when I had a professional problem: COLLEAGUES IN OTHER DEPARTMENTS OF THE HOSPITAL (hierarchy)

130d Moral or personal support when I had a professional problem: OTHER COLLEAGUES IN THE COMMUNITY (hierarchy)

130e Moral or personal support when I had a professional problem: COLLEAGUES AT MY WORKPLACE (hierarchy)

130f. Moral or personal support when I had a professional problem: PROFESSIONAL SOCIETIES OR ORGANIZATIONS IN ROMANIA (hierarchy)

130g Moral or personal support when I had a professional problem: PROFESSIONAL ORGANIZATIONS ABROAD (hierarchy)

130h Moral or personal support when I had a professional problem: SPECIALISTS OF THE HEALTH MINISTRY (hierarchy)

130i. Moral or personal support when I had a professional problem: ONLINE MEDIA INFORMATION GROUPS (hierarchy)

130j. Moral or personal support when I had a professional problem: OTHER SOURCES (hierarchy)

131. My colleagues and I share the same values. [COLLEGIAL RECIPROCITY]

132. How do you rate the trust of your colleagues during the pandemic? [TRUST IN COLLEAGUES].

133. Two weeks before the pandemic, how would you rate the trust of your colleagues? [MUTUAL TRUST AMONG COLLEAGUES] 134.

134. Compared to before the pandemic, I enjoy my work just as much. [WORKING CONDITIONS].

135. Compared to before the pandemic, morale is better where I work. [WORKING CONDITIONS].

136. Compared to before the pandemic, I feel more stressed. [WORKING CONDITIONS] [R]

137. Compared to before the pandemic, I rely more on the support of colleagues. [WORKING CONDITIONS] [R

138. Compared to my work before the pandemic, I rely more on my supervisor for guidance. [WORKING CONDITIONS] 139.

139. The COVID-19 pandemic had a positive effect on the prestige of the medical profession. [PROFESSIONAL PRESTIGE].

140. The COVID-19 pandemic has had a positive effect on my professional prestige. [PROFESSIONAL PRESTIGE].

141. Friends and acquaintances shunned me for fear that I might be infected. [AGENCY] [THREAT]

142. Friends or acquaintances have avoided my partner or children for fear of infection. [AGENCY] [THREAT]

143. My acquaintances have expressed fear that I or one of my family members may have COVID-19. [AGENCY] [THREAT].

144. Would you be willing to answer a similar questionnaire in 6 months?
